# Mitochondrial dysfunction induces radioresistance in colorectal cancer by activating [Ca^2+^]_m_-PDP1-PDH-histone acetylation retrograde signaling

**DOI:** 10.1038/s41419-021-03984-2

**Published:** 2021-09-06

**Authors:** Yingying Shi, You Wang, Huangang Jiang, Xuehua Sun, Hui Xu, Xue Wei, Yan Wei, Guohui Xiao, Zhiyin Song, Fuxiang Zhou

**Affiliations:** 1 Department of Radiation and Medical Oncology, Zhongnan Hospital, Wuhan University, Wuhan, 430071 China; 2 Hubei Key Laboratory of Tumor Biological Behaviors, Zhongnan Hospital, Wuhan University, Wuhan, 430071 China; 3 Hubei clinical cancer study center, Zhongnan Hospital, Wuhan University, Wuhan, 430071 China; 4 Hubei Key Laboratory of Cell Homeostasis, College of Life Sciences, Frontier Science Center for Immunology and Metabolism, Wuhan University, Wuhan, Hubei 430071 China

**Keywords:** Cancer metabolism, Radiotherapy, Double-strand DNA breaks, Predictive markers

## Abstract

Mitochondrial retrograde signaling (mito-RTG) triggered by mitochondrial dysfunction plays a potential role in regulating tumor metabolic reprogramming and cellular sensitivity to radiation. Our previous studies showed phos-pyruvate dehydrogenase (p-PDH) and PDK1, which involved in aerobic glycolysis, were positively correlated with radioresistance, but how they initiate and work in the mito-RTG pathway is still unknown. Our further genomics analysis revealed that complex I components were widely downregulated in mitochondrial dysfunction model. In the present study, high expression of p-PDH was found in the complex I deficient cells and induced radioresistance. Mechanistically, complex I defects led to a decreased PDH both in cytoplasm and nucleus through [Ca^2+^]_m_-PDP1-PDH axis, and decreased PDH in nucleus promote DNA damage repair (DDR) response via reducing histone acetylation. Meanwhile, NDUFS1 (an important component of the complex I) overexpression could enhance the complex I activity, reverse glycolysis and resensitize cancer cells to radiation in vivo and in vitro. Furthermore, low NDUFS1 and PDH expression were validated to be correlated with poor tumor regression grading (TRG) in local advanced colorectal cancer (CRC) patients underwent neoadjuvant radiotherapy. Here, we propose that the [Ca^2+^]_m_-PDP1-PDH-histone acetylation retrograde signaling activated by mitochondrial complex I defects contribute to cancer cell radioresistance, which provides new insight in the understanding of the mito-RTG. For the first time, we reveal that NDUFS1 could be served as a promising predictor of radiosensitivity and modification of complex I function may improve clinical benefits of radiotherapy in CRC.

## Introduction

Colorectal cancer (CRC) is the fourth most deadly cancer with almost 900,000 deaths annually in the world [1]. Radiotherapy is one of the most important treatments for CRC patients. However, intrinsic radioresistance of CRC cells usually leads to recurrence or metastasis in patients who received radiotherapy and results in poor prognosis [2]. Therefore, novel therapeutic strategies for increasing the radiosensitivity of CRC are urgently needed and can be of great value.

Mitochondrial function is often dysregulated in cancer cells, and there is growing interest in exploring how altered mitochondrial function could be targeted to improve radiotherapeutic effects. In a previous study, we found that the mitochondrial DNA (mtDNA)-reduced cells showed a switch to aerobic glycolysis and exhibited more resistance to chemotherapy in CRC [3]. Our data also revealed that mtDNA depletion might activate the mitochondria-to-nucleus retrograde signaling pathway and induce radioresistance in H1299 cells [4]. As mitochondrial respiration defects are supposed to be the underlying basis for aerobic glycolysis in cancer [5], and functional mitochondrial transplantation has been reported to rescue mitochondrial respiration and significantly enhanced radiosensitivity in glioma [6], we then wonder whether mitochondrial respiration defects contribute to radioresistance. Thus, we performed transcriptomics analysis to identify mitochondrial complex genes in the mitochondrial dysfunction model, interestingly, we found that the complex I components were widely downregulated in mtDNA depletion cells. Recent study also revealed that the degradation of complex I components (NDUFB5, NDUFB6) participated in glycolysis and promote CRC development [7].

Complex I acts as the rate-limiting step in overall electron transfer and plays a central role in mitochondrial oxidative respiration [8]. Meanwhile, complex I is also required for mitochondrial Ca^2+^ ([Ca^2+^]_m_) homeostasis [9]. Furthermore, mitochondrial Ca^2+^-uptake protein MICU1-silenced cells could elevate basal [Ca^2+^]_m_ and PDH expression and overcome chemotherapy resistance [10], that was because [Ca^2+^]_m_ was necessary for PDP1 activation [11], which dephosphatased p-PDH to PDH [12]. More and more metabolic enzymes or metabolites have shown their non-metabolic regulatory effects [13, 14]. Our previous study also showed that Dichloroacetate could lead to the reactivation of PDH and increased DNA damage induced by irradiation [15]. However, the exact effects of downregulated PDH on radiosensitivity are poorly understood. PDH existing in the nucleus has been reported to promote histone acetylation modification and regulate gene transcription [16]. Radiotherapy’s cytotoxic effect is mainly exerted by inducing DNA double-strand breaks (DSBs) [17]. But the compensatory mechanisms like DNA damage response in cancer cells can result in resistance to radiotherapy [18]. Histone deacetylases (HDACs) participate in DNA repair [19, 20] and HDAC1/ HADC2 contributes to the repair of DSBs via down-regulating the H3K56 and H3K9 acetylation [21, 22]. Thus, we hypothesize that the downregulated PDH caused by complex I defects may promote DNA repair and then cause radioresistance.

Based on previous study, we explore the relationship between PDH and radiosensitivity in mitochondrial dysfunction cell models. The results indicated that the complex I defects induce radioresistance by activating [Ca^2+^]_m_-PDP1-PDH-histone acetylation retrograde signaling in vitro and in vivo. That low NDUFS1 and PDH are associated with radioresistance is further confirmed in clinical pathological tissues. Therefore, we report a novel role of complex I in metabolic reprogramming accounting for radioresistance in CRC.

## Results

### Complex I components were downregulated in radioresistant cells and tumor tissues

**Fig. 1:**
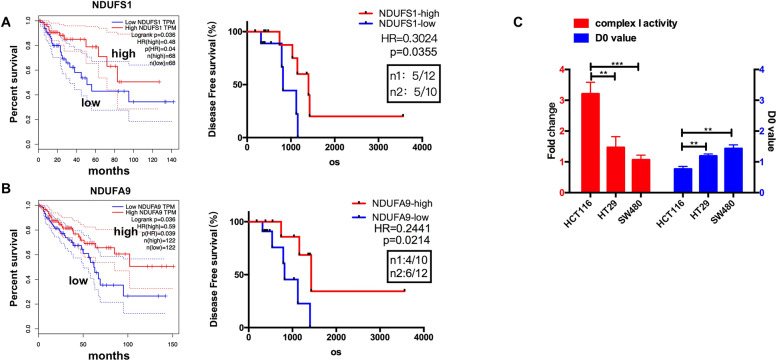
Complex I components were downregulated in radioresistant cells and tumor tissues. **A**–**B** Kaplan–Meier curves showing the overall survivals and disease-free survivals in colorectal tumor cases with low or high NDUFS1 and NDUFA9. **C** Mitochondrial complex I activity in HCT116, HT29, and SW480 cells were shown in the left, and the D0 value of them were shown in the right. Data are presented as the means ± s.e.m. (*n* = 3), **P < 0.01, ***P < 0.001.

The mitochondrial dysfunction cell models (H1299 ρ^0^) constructed previously displayed resistance to radiation, compared with the parental ρ^+^ cells [4]. Based on mitochondrial respiration is suppressed in cancer cells due to mitochondrial dysfunction, and our further results of genomics analysis about H1299 ρ^0^ and ρ^+^ cells suggesting that the complex I genes were widely downregulated in ρ^0^ cell models (Table 1), we proposed that complex I might involve in radiosensitivity. It has been reported that the loss of functional complex I is a candidate driver event in kidney cancer [23]. We also found complex I genes listed in Table 1 (NDUFS1, NDUFA9, NDUFA10) were positively associated with overall survival, as well as disease-free survival after radiotherapy in CRC (Fig. 1A, B and Supplemental Fig. 1). These results demonstrated that complex I genes were downregulated in radioresistant cells and tumor tissues, making it worthy of further investigation. In order to verify the correlation between complex I function and radiosensitivity in CRC, we examined the activity of complex I in CRC cell lines with different intrinsic radiosensitivity. SW480 reported as the highly radioresistant cell lines (highest D0 value) had greater decreases in complex I activity (Fig. 1C), while HCT116 (lowest D0 value) had the highest level of complex I activity, and HT29 was in the middle, meaning that the complex I defects in radioresistant cells.

**Table 1:** Identification of downregulated genes of complex I in H1299 ρ^0^ and ρ^+^ cells.

Complex I			
GeneID	fc value	Symbol	Name
4719	0.633	NDUFS1	NADH dehydrogenase (ubiquinone) Fe-S protein 1
4720	0.669	NDUFS2	NADH dehydrogenase (ubiquinone) Fe-S protein 2
4724	0.606	NDUFS4	NADH dehydrogenase (ubiquinone) Fe-S protein 4
4725	0.871	NDUFS5	NADH dehydrogenase (ubiquinone) Fe-S protein 5
4726	0.501	NDUFS6	NADH dehydrogenase (ubiquinone) Fe-S protein 6
4695	0.819	NDUFA2	NADH dehydrogenase (ubiquinone) 1 alpha subcomplex 2
4696	0.850	NDUFA3	NADH dehydrogenase (ubiquinone) 1 alpha subcomplex 3
4698	0.699	NDUFA5	NADH dehydrogenase (ubiquinone) 1 alpha subcomplex 5
4702	0.719	NDUFA8	NADH dehydrogenase (ubiquinone) 1 alpha subcomplex 8
4704	0.709	NDUFA9	NADH dehydrogenase (ubiquinone) 1 alpha subcomplex 9
4705	0.652	NDUFA10	NADH dehydrogenase (ubiquinone) 1 alpha subcomplex 10
4723	0.863	NDUFV1	NADH dehydrogenase (ubiquinone) flavoprotein 1
4729	0.648	NDUFV2	NADH dehydrogenase (ubiquinone) flavoprotein 2
4711	0.806	NDUFB5	NADH dehydrogenase (ubiquinone) 1 beta subcomplex 5

### Complex I deficient cells exhibited radioresistance and enhanced DNA damage repair

**Fig. 2:**
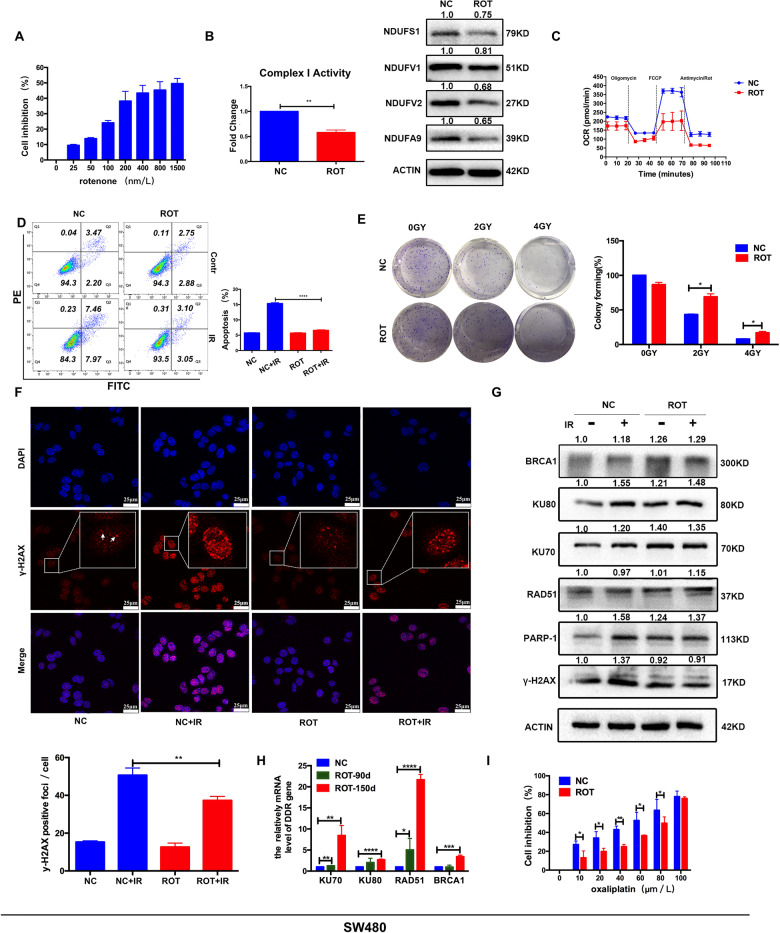
Complex I deficient cells exhibited radioresistance and enhanced DNA damage repair. **A** Cell inhibition rate of SW480 cells treated with various concentrations of rotenone for 24 h. **B** Relative mitochondrial complex I activity and complex I protein in NC and ROT cells. The band intensity was quantified with Image lab software (Bio-Rad) and normalized to Actin. (*n* = 3), **P < 0.01. **C** Oxygen consumption rate (OCR) was measured in NC and ROT cells. **D** Apoptosis was quantified by combined staining of annexin V and PI, and fluorescence was analyzed using flow cytometry. (*n* = 3), ****P < 0.0001. **E** Clonogenic potential with 0 Gy, 2 Gy, 4 Gy irradiation and statistical analysis. (*n* = 4), *P < 0.05. **F** After 4 Gy X-ray irradiation, confocal microscopy was used to visualize the intracellular γ-H2AX foci (red) and DAPI (blue) in NC and ROT cells. Inset shows magnified areas, and the white arrow indicates foci. Scale bar: 25 μm. Foci per cell was calculated, (*n* = 6), **P < 0.01. **G** The DNA damage repair protein in NC and ROT cells with or without 4 Gy X-ray irradiation. **H** mRNA levels of DDR genes in NC and ROT cells at different periods (70 days and 150 days) were analyzed by real-time PCR. (*n* = 5), **P < 0.01, ***P < 0.001, ****P < 0.0001. **I** Cell inhibition rate of different concentrations of oxaliplatin on NC and ROT cells for 24 h (*n* = 5), *P < 0.05, **P < 0.01. Data are presented as the means ± s.e.m.

To carry out the next study bearing current understanding, we constructed a complex I deficient cell model by exposing to rotenone for continuous subculture. Rotenone has been recognized as an inhibitor of complex I [24]. Due to the cytotoxic effects of rotenone, the optimal concentration was first explored by CCK8 assay (Fig. 2A). We choosed 50 nm/L of rotenone for the subsequent experiments, which was the concentration required for partial inhibition of complex I. The complex I deficient cells were named as SW480-Rot (ROT). Compared with the parental SW480 cells (NC), decreased complex I activity and complex I proteins expression were observed in ROT cells (Fig. 2B), as well as oxygen consumption rate (OCR), a major biochemical parameter indicating mitochondrial respiration (Fig. 2C). The decrease in OCR might result from complex I defects, which suggests that complex I indeed play an important role in energy metabolism.

To clarify the function of complex I in radiosensitivity, apoptosis of NC and ROT cells treated with or without X-ray were analyzed. At 48 h post-irradiation, the apoptotic rate decreased to 6.1% in ROT cells, compared to 15.4% in NC cells (Fig. 2D). While the colony-forming ratio of ROT cells treated with X-ray irradiation was much more higher than that of NC cells treated with X-ray (Fig. 2E), which ruled out the effect of rotenone on the proliferation (Supplemental Fig. 2a). These results revealed that complex I deficient cells exhibited radioresistance. To further investigate the radiobiological effects of complex I defects, radiation-induced DNA double-strand break was measured by the staining of γ-H2AX. There were decreased γ-H2AX foci (Fig. 2F) and increased DNA damage repair proteins in SW480-rot cells treated with or without irradiation compared with SW480 cells (Fig. 2G, H). Besides, mRNA levels of DDR genes were also higher than SW480 cells, and the longer the time exposed to rotenone, the higher mRNA levels of DDR genes in ROT cells. In addition, we also noticed that ROT cells were less sensitive to oxaliplatin in CCK8 assay (Fig. 2I) and apoptosis analysis (Supplemental Fig. 2b), indicating enhanced capability of DNA repair in ROT cells. Taken together, it is concluded that the increased DNA damage repair in ROT cells may lead to radioresistance.

### Downregulation of PDH in complex I deficient cells contributes to glycolysis and DNA repair

**Fig. 3:**
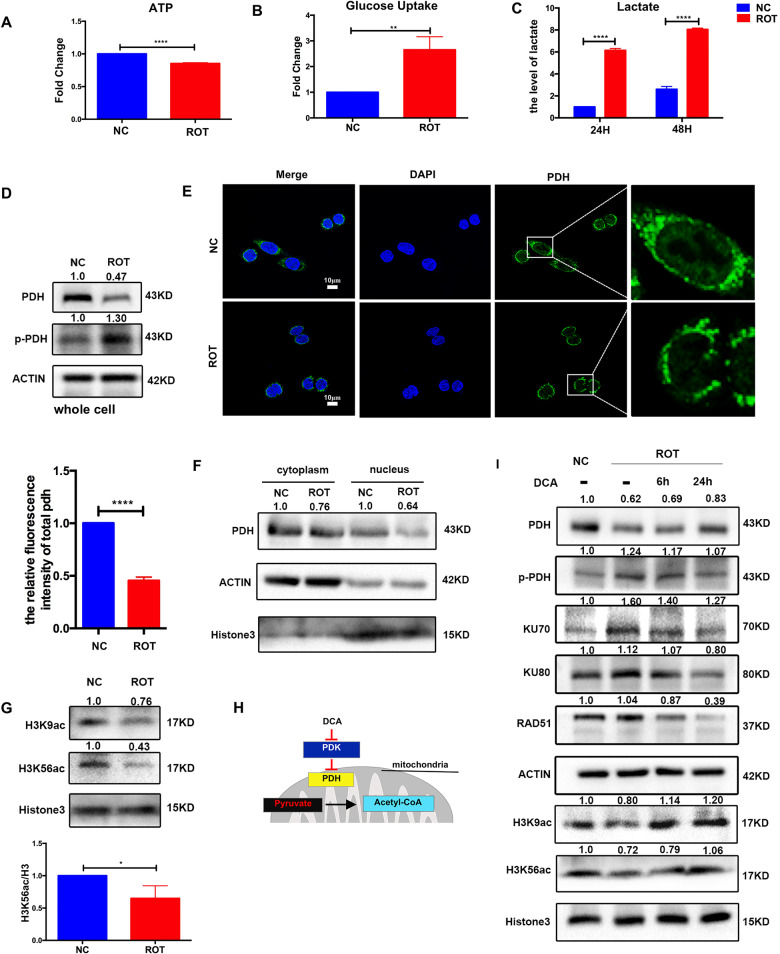
Downregulation of PDH in complex I deficient cells contributed to glycolysis and DDR. **A**–**C** Complex I deficient cells showed decreased ATP level (**A**) and increased glucose uptake (**B**), lactate secretion (**C**) compared with that in control cells. (*n* = 3), **P < 0.01, ****P < 0.0001. **D** Levels of PDH and p-PDH protein in NC and ROT cells. **E** Confocal photography showed the levels of PDH (green) and DAPI (blue) in the cytoplasm and nucleus in NC and ROT cells. Magnified areas taken at ×5 magnification, scale bar: 10 μm. Relative fluorescence intensity was shown. (*n* = 5), ****P < 0.0001. **F** Study of PDH expression in extracted nucleus and cytoplasm of NC and ROT cells by western blot. **G** Levels of H3K9ac and H3k56ac protein in NC and ROT cells, Relative protein levels of H3K56ac were normalized to H3 (*n* = 3), *P < 0.05. **H** Mechanism for DCA-mediated increase in histone acetylation. **I** Western blot analysis of PDH, H3K9ac, H3k56ac, DNA repair protein in NC cells and ROT cells with or without DCA (5 mM) for 6 h and 24 h in ROT group. Data are presented as the means ± s.e.m.

Apart from being the key enzyme of glycolysis in cytoplasm, PDH in the nucleus was reported to be responsible for histone acetylation and histone hypoacetylation promotes DNA repair [25]. As expected, ROT cells showed increased glycolysis: decreased ATP (Fig. 3A) and significantly increased level of glucose uptake and lactate (Fig. 3B, C), which were three major biochemical parameters for glycolytic activity. Besides, ROT cells showed lower PDH and higher p-PDH expression compared with NC cells (Fig. 3D). These results indicated that complex I deficiency induced a metabolic switch that drives aerobic glycolysis. Moreover, the confocal microscopy and western blot results of isolated nuclei and cytoplasm showed that the PDH both in the cytoplasm and nucleus decreased (Fig. 3E, F), consistent with the level of H3K9 and H3K56 acetylation in ROT cells (Fig. 3G). We speculated that diminished PDH in the nucleus led to decrease of histone acetylation. To determine whether the reduction of H3K9 and H3K56 acetylation were mediated by downregulation of PDH, we performed the following experiment to activate PDH (Fig. 3H) and elevated PDH expression was observed in ROT cells after adding dichloroacetate (DCA). DCA treatment restored H3K9, H3K56 acetylation in a dose-dependent manner, accordingly, the DNA repair protein expression decreased (Fig. 3I). In brief, we have revealed that complex I defects induce the downregulation of PDH, which drives metabolic switch to glycolysis and reduced histone acetylation.

### Complex I deficiency activated the [Ca^2+^]_m_-PDP1-PDH-histone acetylation retrograde signaling pathway

**Fig. 4:**
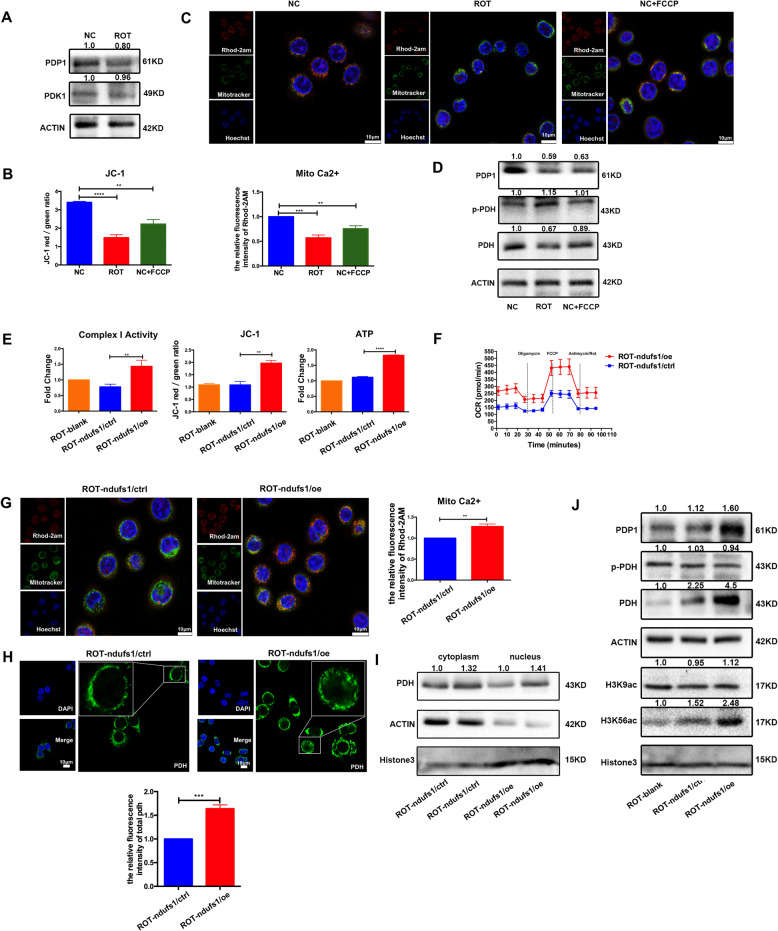
Complex I deficiency activated the [Ca^2+^]m–PDP1-PDH-histone acetylation retrograde signaling pathway. **A** Western blot analysis of PDK1 and PDP1 protein in NC and ROT cells. **B** Statistical analysis of ΔΨm in NC, NC + FCCP (10 μM), ROT cells. (*n* = 3), **P < 0.01, ****P < 0.0001. **C** The above cells were incubated with the fluorescent dyes Rhod-2AM (5 μM, red), Mitotracker (200 nM, green) and Hoechst (2 μg/ml, blue), rotenone and FCCP caused a rapid decrease in [Ca^2+^]_m_. Scale bar:10 μm. Relative fluorescence intensity was shown, (*n* = 3), **P < 0.01, ***P < 0.001. **D** Levels of PDP1, p-PDH, and PDH protein in NC, NC + FCCP, and ROT cells. **E**–**F** Overexpression of NDUFS1 could increase complex I activity, oxygen consumption, ATP and the ΔΨm. (*n* = 3), **P < 0.01, ****P < 0.0001. **G** [Ca^2**+**^]_m_ level in ROT-ndufs1/ctrl and ROT-ndufs1/oe cells. Scale bar:10 μm. Relative fluorescence intensity was shown, (*n* = 3), **P < 0.01. **H** IF staining of PDH in ROT-ndufs1/ctrl and ROT-ndufs1/oe cells, scale bar:10 μm. Relative fluorescence intensity was shown, (*n* = 5), ***P < 0.001. **I** Levels of PDH expression in extracted nucleus and cytoplasm in ROT-ndufs1/ctrl and ROT-ndufs1/oe cells. **J** Western blot analysis of PDP1, p-PDH, PDH and histone acetylation protein in ROT-balnk, ROT-ndufs1/ctrl and ROT-ndufs1/oe cells. Data are presented as the means ± s.e.m.

The corresponding increase in p-PDH expression in ROT cells suggests that PDH is regulated by phosphorylation modification, we then detected the levels of pyruvate dehydrogenase kinase (PDK1) and PDP1 in NC and ROT cells to identify the intracellular signaling pathway. Western blot showed that PDP1 was lower in ROT cells, while PDK1 had no obvious change (Fig. 4A). We next explored the molecular mechanism, the level of mitochondrial ROS generation in NC and ROT cells were first examined. As shown in Supplemental Fig. 2c, there was an increased ROS production, but N-Acetyl-L-cysteine (NAC 5 mM, a ROS scavenger) could not increase the apoptosis in ROT cells, at 48 h post-irradiation. In this context, [Ca^2+^]_m_ was supposed to be required for PDP1 activation [11, 26] and the process of the mitochondrial Ca^2+^ uptake pathway was driven by ΔΨm (Supplemental Fig. 2d). In fact, complex I indeed provide a basis for tumor Calcium homeostasis which involved in RTG [27, 28]. We hypothesized that complex I deficiency might inhibit PDP1 activity by decreasing Ca^2+^-overload in the mitochondria. Next, we detected the level of mitochondrial membrane potential in NC and ROT cells. As shown there was a significant ΔΨm loss in complex I deficient cells (Fig. 4B). In addition, ROT cells also had decreased basal [Ca^2+^]_m_ as compared to the NC cells using rhod-2am fluorescence-based mitochondrial Ca^2+^ studies [29] (Fig. 4C). Furthermore, FCCP (the mitochondria-specific ionophore that can induce disrupted ΔΨm. Fig. 4B), was used to decrease mitochondrial Ca^2+^ uptake in SW480 (Fig. 4C). And we found that PDP1, PDH expression were also decreased in the FCCP group (Fig. 4D), suggesting that reduced PDP1 expression might result from the restriction of mitochondrial Calcium uptake.

To directly test whether complex I is functionally important for this pathway, we constructed a stably NDUFS1-overexpressed ROT cell lines (ROT-ndufs1/oe) to restore complex I activity. The transfection efficiency of NDUFS1 was verified by qPCR and western blotting (Supplemental Fig. 3a, b). As shown in Fig. 4E and F, NDUFS1 overexpression increased the complex I activity, ATP, and OCR levels compared with that in control cells (ROT-ndufs1/ctrl). Furthermore, ROT-ndufs1/oe cells restored [Ca^2+^]_m_ level, and PDH protein expression by elevating ΔΨm, as compared to ROT-ndufs1/ctrl cells (Fig. 4G, H). Histone acetylation also raised in ROT-ndufs1/oe cells (Fig. 4J) as a result of the increased PDH in the nucleus (Fig. 4I). Overall, the above results indicated that the NDUFS1 overexpression could rescue the activity of complex I and promote mitochondrial respiration in ROT cells, which led to increased ΔΨm and mitochondrial Calcium uptake, suggesting complex I defects may result in radioresistance via [Ca^2+^]_m_-PDP1-PDH-histone acetylation retrograde signaling pathway.

### Enhancing complex I activity induced by NDUFS1 overexpression sensitized CRC cells to radiation

**Fig. 5:**
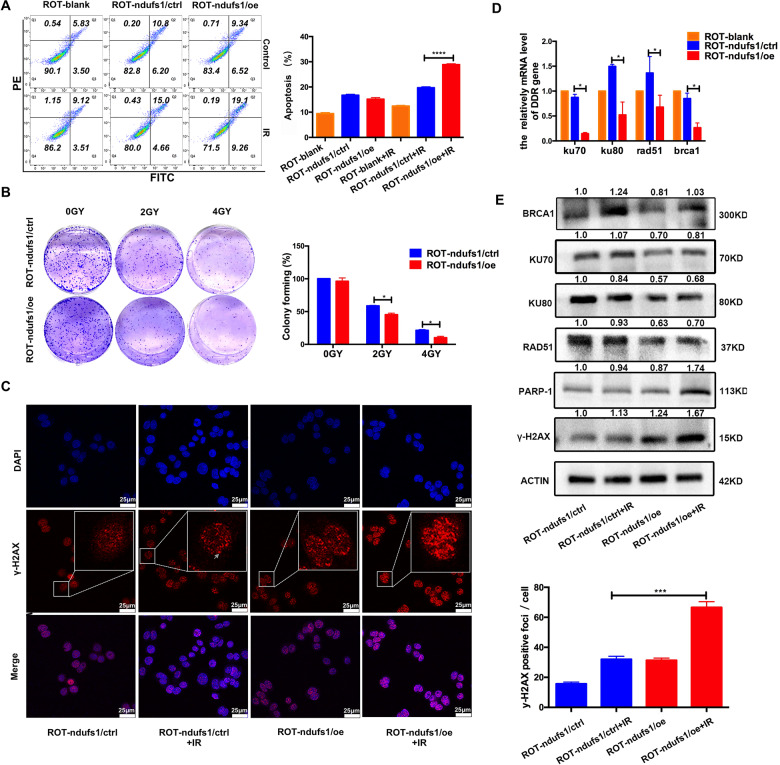
Enhancing mitochondrial complex I activity induced by NDUFS1 overexpression sensitized CRC cells to radiation. **A**, **B** Apoptosis (**A**) and colony formation assay (**B**) in ROT-ndufs1/ctrl and ROT-ndufs1/oe cells with or without 4 Gy X-ray irradiation. (*n* = 4), *P < 0.05, ****P < 0.0001. **C** IF staining of γ-H2AX foci (red) and DAPI (blue) in ROT-ndufs1/ctrl and ROT-ndufs1/oe cells with or without 4 Gy X-ray irradiation. Inset shows magnified areas, scale bar: 25 μm. Foci per cell was calculated, (*n* = 6), ***P < 0.001. **D**–**E** Overexpression of NDUFS1 could decrease mRNA and protein level of DDR genes. (*n* = 5), *P < 0.05, Data are presented as the means ± s.e.m.

As NDUFS1 overexpression can enhance mitochondrial metabolism, functional assays were then perfomed to investigate its role in regulating radiosensitivity. From this analysis, we noted that the apoptosis and γ-H2AX foci were increased in the NDUFS1-overexpression cells at post-irradiation, compared with the control cells. (Fig. 5A, C). While decreased colony formation was also observed in NDUFS1-overexpression cells treated with various dose X-ray irradiation (Fig. 5B). This phenomenon that CRC cells became sensitive to radiation was accompanied with the decreased mRNA and protein level of DNA repair associated molecules (Fig. 5D, E), which supported the notion that overexpression of NDUFS1 suppressed DNA repair and improved radiosensitivity. In conclusion, our research fully reveals that complex I defects lead to radioresistance and NDUFS1 overexpression can restore radiosensitivity in CRC by modulating the [Ca^2+^]_m_-PDP1-PDH-histone acetylation axis.

### NDUFS1-overexpression increased radiosensitivity in xenograft model

**Fig. 6:**
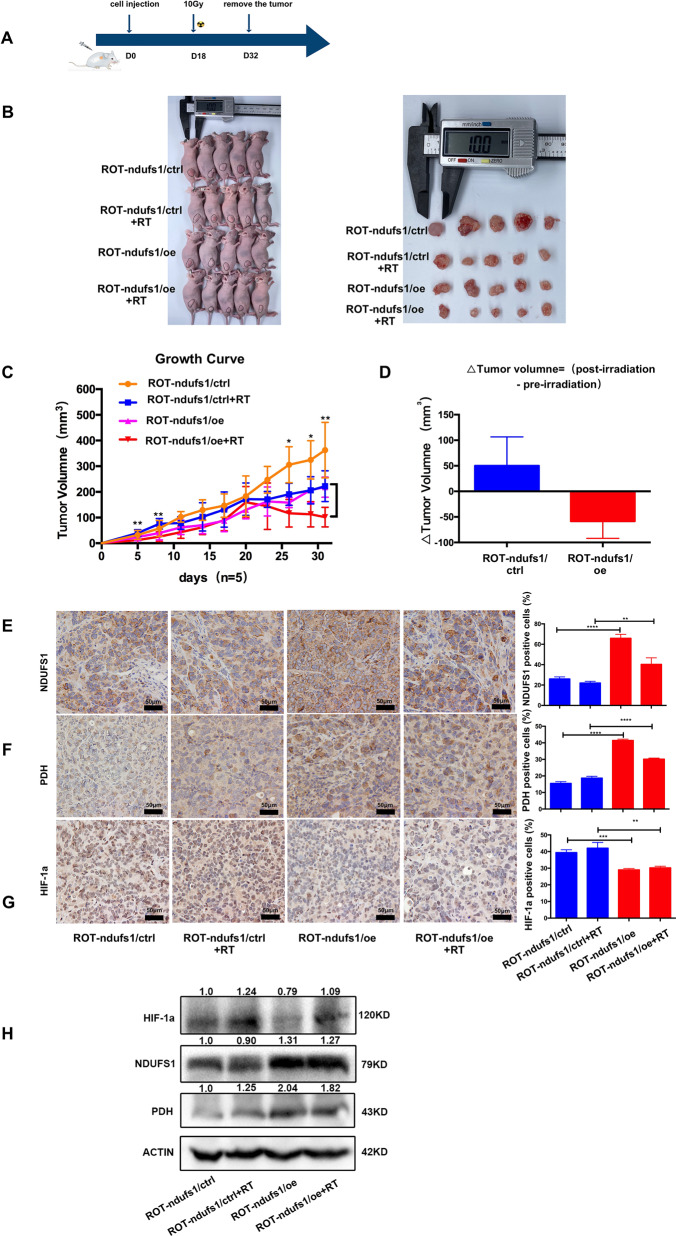
NDUFS1-overexpression increased radiosensitivity in xenograft model. **A** Treatment schema. **B** Tumor images after all the mice were killed and tumors were removed. **C** Tumor size was measured every 3 days, control+ irradiation vs oe + irradiation, (*n* = 5), *P < 0.05 **p < 0.01. **D** △Tumor Volume = (post-irradiation-pre-irradiation) in ROT-ndufs1/ctrl and ROT-ndufs1/oe groups. **E** Representative images of IHC staining of NDUFS1in different tumor tissues. Brown: NDUFS1-positive cells. (*n* = 3), **P < 0.01, ***P < 0.001. **F** IHC staining of PDH in different tumor tissues. (*n* = 3), ****P < 0.0001. **G** IHC staining of HIF-1α in different tumor tissues. (*n* = 3), **P < 0.01, ***P < 0.001. **H** Expression of NDUF1, PDH, HIF-1a in tumor tissues by western blot. Data are presented as the means ± s.e.m.

We next explored the radiosensitization of NDUFS1 overexpression in vivo, using ROT-ndufs1/ctrl and ROT-ndufs1/oe cells for xenotransplantation studies (Fig. 6A). Consistent with in vitro experiment (Supplemental Fig. 3d), the xenograft tumor volume in the ROT-ndufs1/oe was significantly smaller than that in the ROT-ndufs1/ctrl group (Fig. 6B, C). Furthermore, compared to the growth delay caused by irradiation alone, NDUFS1 overexpression combined with X-ray irradiation directly shrunk the tumor size in nude mice suggesting a radiosensitization of NDUFS1 overexpression (Fig. 6D). Another consequence of NDUFS1 overexpression (Fig. 6E) in mice model was a higher PDH (Fig. 6F) and lower hypoxia-inducible factor 1a (HIF-1α) expression (Fig. 6G) in the IHC results, which indicated the decline in glycolysis. In addition, the western blot results of tumor tissues also showed higher PDH and lower HIF-1α in NDUFS1 overexpressing groups combined with or without irradiation (Fig. 6H). Our preclinical results demonstrate that the enhancement of complex I can overcome the radioresistance in hypoxic tumors or glycolysis dominant tumors due to mitochondrial dysfunction.

### Elevated NDUFS1 and PDH expression were significantly associated with better tumor regressions in patients with CRC

**Fig. 7:**
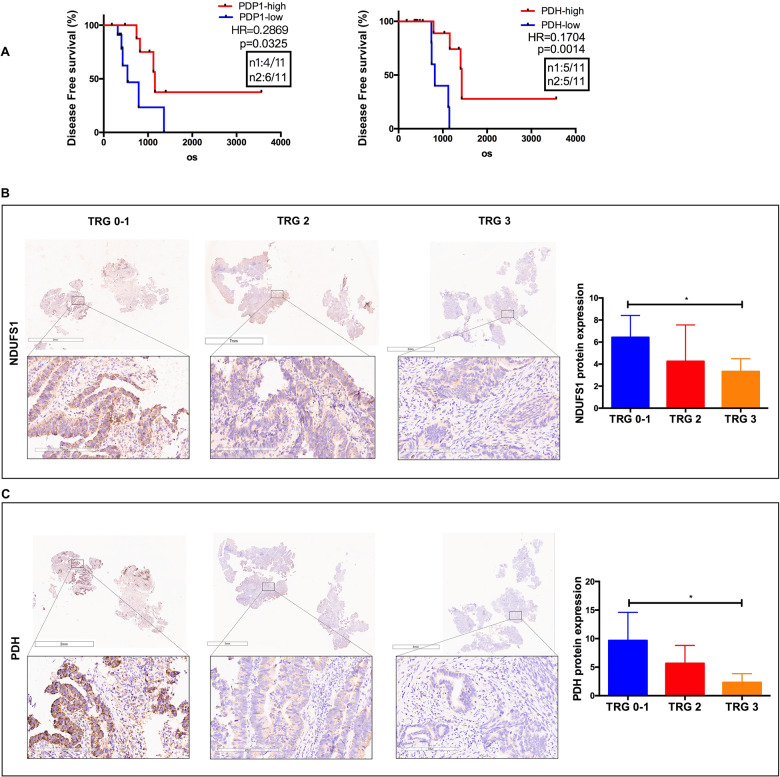
Elevated NDUFS1 and PDH expression were significantly associated with better tumor regressions in patients with CRC. **A** Kaplan–Meier curves showing the disease-free survivals in colorectal tumor cases with low or high PDH, PDP1 expression from TCGA databases. **B**–**C** IHC analysis of NDUFS1 and PDH protein expression in patients with different TRG score. Magnified areas were shown below, scale bars :2–7 mm. NDUFS1 and PDH expression was lower in worse TRG tissues. (*n* = 3), *P < 0.05. Data are presented as the means ± s.e.m.

To provide robust support for our findings, we used clinically annotated mRNA data from TCGA (The Cancer Genome Atlas) databases. The mRNA expression data for all genes were detected by microarray. The Kaplan–Meier survival analysis revealed that low expression of NDUFS1 (Fig. 1A), PDP1(rather than PDK1), and PDH, was correlated with poor prognosis respectively in colorectal cancer patients who received radiotherapy (Fig. 7A and Supplemental Fig. 4a). We also analyzed the expression of NDUFS1 and PDH in biopsy tissues collecting from patients underwent preoperation radiation in Zhongnan Hospital. The tumor regression grading (TRG) after neoadjuvant radiotherapy was collected to evaluate the efficacy of radiotherapy. The clinicopathological features of patients were as follows (Table 2). TRG was stratified according to the four-point score system reported by the 8th edition of the American Joint Committee on Cancer (AJCC) Cancer Staging Manual. Of note, cases with higher expression of NDUFS1 and PDH were significantly associated with lower TRG score (Fig. 7B, C). NDUFS1 and PDH may be potential indictors of radiosensitivity for patients with CRC. These results support our hypothesis that complex I defects drive aerobic glycolysis and radiation resistance in CRC.

**Table 2:** Clinicopathological features of CRC patients (*n* = 15).

Clinicopathological feature	Ages (years)		Gender		Patheological type		TRG score		
	≤60	>60	Male	Female	Adenocarcinoma	High-grade intraepithelial neoplasia	0–1	2	3
Case (*n*, %)	13 (87)	2 (13)	9 (60)	6 (40)	14 (93)	1 (7)	6 (40)	6 (40)	3 (20)

## Discussion

Mitochondrial retrograde signaling (mito-RTG) had been described and characterized in metastasis and chemoresistance of many human cancers, but few studies referred to the role of mito-RTG in regulating cellular sensitivity to radiation [30–32]. We previously observed that the radioresistant cell lines exhibited mitochondrial dysfunction, and mitochondrial malfunctions of cancer cells also induced radioresistance [4], which led us to investigate how mito-RTG manipulated in the crosstalk between mitochondria and nucleus in the cellular response to radiation. We then performed genomics analysis to discover that complex I components were widely downregulated in mitochondrial dysfunction model. Complex I is known as the largest complex in mitochondrial respiratory chain and its deficiency caused by complex I gene mutation is supposed to be the culprit of mitochondrial dysfunction. Unsurprisingly, in this study we found complex I deficient cancer cells showed increased resistance to radiation as well as enhanced glycolysis, which, to some extent, consistent with our earlier findings mentioned above.

Glycolysis had been reported to promote radioresistance [30] and the inhibition of glycolysis mainly increased redox metabolism and resulted in DSB. But how the exact effect of glycolysis on DSB remains to be elucidated. In consideration of the essential role of PDH in glycolysis [31], and the inhibition of PDK1/ p-PDH activity with DCA was found to improve radiosensitivity previously [15], we further explore the mito-RTG involving PDH in complex I defects model and revealed decreased PDH in cytoplasm and nucleus in complex I deficient cells. Mechanistically, the ΔΨ loss results from complex I defects lead to a decrease in mitochondrial calcium [32], and the decreased [Ca^2+^]_m_ cause inactivation of PDP1 [33]. Notably, the PDH in cytoplasm and nucleus are decreased in complex I deficient cells, which are different from the phenomenon that rotenone caused increased nuclear PDH combined with serum and rhEGF [16]. Actually, studies have demonstrated that PDP1 (but not PDPII) is also present in the cytoplasm and nucleus. Thus, we suppose that PDH is decreased both in cytoplasm and nucleus as a result of PDP1 inactivation. Besides, that the translocation of PDH into the nucleus was promoted by rotenone was based on serum and rhEG, whereas the effect of rotenone alone was not clear. The results of confocal microscopy and nuclear protein extraction support our hypothesis. On the other hand, decreased PDH in nucleus promote DNA damage repair via reducing histone acetylation [34, 35]. The DNA repair gene can be regulated by histone acetylation [36] and the inhibition of DNA repair have been showed to increase radiosensitivity [37]. DNA damage repair occurs primarily via the error-prone non-homologous end-joining (NHEJ) or the homologous recombination (HR) pathway. NHEJ works by activating ATM, and the Ku heterodimer [38], while HR involves BRCA1, RAD51, etc. Our study indicates that proteins involved in both two pathways are increased, strongly suggesting that the enhanced DNA damage repair is result from transcription regulation rather than a simple protein modification.

NDUFS1 is an accessory subunit of the complex I and belongs to NADH dehydrogenase module. Low NDUFS1 expression has been reported to be correlated with poor prognosis in non-small cell lung cancer [39]. Furthermore, mutation of NDUFB9 (NADH dehydrogenase (ubiquinone) 1 beta sub-complex, 9) might lead to complex I deficiency, and promote tumor metastasis [40]. Up to now, no study has reported NDUFS1 involved in radiosensitivity in CRC. Here we reveal that NDUFS1 overexpression could enhance the complex I activity, reverse glycolysis and resensitize cancer cells to radiation in vivo and in vitro. Contrast to a study in ductal adenocarcinoma (PDAC), targeting to inhibit mitochondrial complex I function has been reported to be anti-tumor [41]. Likely, owing to highly rely on oxidative phosphorylation in PDAC [42], the pancreatic cancer cells may be more sensitive to the inhibition of mitochondrial respiration. Therefore, our finding that complex I deficiency promotes radioresistance and NDUFS1 overexpression reverses this process may consider intratumor metabolic heterogeneity.

Moreover, NDUFS1 overexpression can not only significantly reduce the volume of xenografted tumors after radiotherapy, but also directly inhibit tumor growth in nude mice. Recent study also shows that reintroducing PDSS2 into hepatocellular carcinoma can improve mitochondrial complex I activity and inhibits tumor progression in nude mice [43]. We guess it may be related to decreased glycolysis and the improvement of hypoxia. It has been reported that glycolysis allows the cell to meet requirements for proliferation [44] and HIF-1α facilitates the growth of new blood vessels. Meanwhile, loss of HIF-1α in cancer cells was found to slow xenograft tumors [45]. Moreover, the degradation of complex I components was recently reported to stabilize HIF-1α [7], and enhanced mitochondrial respiration was dispensable for preventing the hypoxia [46], which may provide evidence for the decreased HIF-1α resulting from NDUFS1 overexpression.

In addition, low NDUFS1 and PDH expression were validated to be associated with poor prognosis and TRG score in CRC patients who received neoadjuvant radiotherapy. In previous study, it was also reported that higher p-PDH in cervical cancer was related to the worse efficacy of radiotherapy. To further explore the role of complex I genes on the prognosis of patient with cervical cancer, the tissue microarray (TMA) containing 167 cervical cancer samples was performed, in which 84 treated with radiotherapy were analyzed. Interestingly, cases with lower NDUFA9 rather than NDUFS1 expression have a higher incidence of recurring events after radiotherapy (Supplemental Fig. 4b). As NDUFA9 is also an important subunit of complex I, the importance of complex I and PDH in radiosensitivity were confirmed by our above data. Nevertheless, our data suggest that different genes play a dominant role in complex I function in different cancers, which remains further studies.

Overall, our study demonstrated the [Ca^2+^]_m_-PDP1-PDH-histone acetylation axis activated by complex I defects contribute to intrinsic radioresistance in cancer cell. Moreover, complex I component NDUFS1 could be identified as a new predictive mark of radiosensitivity in CRC, which suggests targeting enhancement of complex I function will be an attractive strategy to improve radiosensitivity.

## Materials and methods

### Cell culture and developing mitochondrial respiratory defect model

SW480 cell line, derived from human colon cancer, was purchased from China Center for Type Culture Collection and were authenticated (Supplemental materials). They were cultured in DMEM HG supplemented with 10% FBS. The “functional defect” condition was generated by exposing the SW480 clone to sub-cytotoxic doses of complex I inhibitor rotenone (MCE HY-B1756) for continuous subculture, as described [24].

### Cell transfection

For NDUFS1 overexpression, SW480-rot cells were transduced with lentiviral particles (LV6 Lentiviral Transduction Particles–Gene Pharma OE LV6-NDUFS1-homo) and 48 h after transduction selected with 6 μg/mL puromycin.

### Chemicals and reagents

Cisplatin (MCE HY-17394) were used as cytotoxic pharmacologic agents. DCA (MCE HY-Y0445A) were used to activate PDH. FCCP (MCE HY-100410) were used to decrease the mitochondrial membrane potential.

### Antibodies

Primary antibodies against NDUFS1 (no. 12444), NDUFA9 (no. 20312-1-AP), NDUFV1 (no. 11238), NDUFV2 (no. 5301), KU80 (no. 6389), KU70 (no. 10723), RAD51 (no. 14961), PARP-1 (no. 13371), PDP1 (no. 21176),PDK1 (no. 10026), β-actin (no. 60008), and Histone3 (no.17168) were obtained from Proteintech and γ-H2AX (no. AP0099), PDHA1 (no. A17432) were obtained from Abclonal and p-PDH (no.ab115343), HIF1a (no. ab179483) were obtained from Abcam, and Acetyl-Histone H3 (Lys9) (no. 9649 s), Acetyl-Histone H3 (Lys56) (no. 4243 s), were obtained from CST in this study.

### Mitochondrial complex I assay

The activity of mitochondrial complex I defined as the oxidation of NADH to NAD+, was determined using the complex I enzymatic activity assay kit (Nanjing jiancheng, A089-1-1) as recommended by the manufacture.

### Mitochondrial membrane potential

To detect mitochondrial membrane potential, cells were stained with JC-1 dye (Biyotime C2006) for 20 min at 37 °C. The fluorescence intensity was determined by flow cytometry (beckman) as an index of the mitochondrial membrane potential.

### Metabolic analysis

Cellular ATP was measured using an ATP assay kit (Beyotime S0026) according to the manufacturer’s protocol and was normalized to the cell protein content. Lactate production was measured with a Lactate Assay Kit (Nanjing jiancheng A019-2-1). Cell culture medium was incubated with a lactate assay buffer containing lactate enzyme for 30 min. Then were measured with a Microplate Reader (BioTek) at 570-nm wavelength. The OCR was measured at 37 °C with an XF96 instrument (Seahorse Bioscience). Cells were sequentially treated with 0.5 mM Oligomycin, 2 mM FCCP and 1 mM Antimycin + Rotenone (A + R) at indicated time points.

### ROS assay

For ROS assay, cells were stained with the 10 μm fluorescence probe DCFH-DA (Biyotime S0033S) at 37 °C for 30 min. The fluorescence intensity was determined by flow cytometry (beckman).

### Quantitative reverse transcription PCR

Total RNA was extracted with the RNeasy Mini Kit and reverse transcribed using the Prime Script RT Reagent Kit. Subsequently, real-time PCR was performed with SYBR Premix Ex Taq (above all purchase from Aidlab Biotechnologies) using an ABI Prism 7900 instrument (Applied Biosystems).

The primer sequences used in this study are as follows:

ACTIN: F 5′-GAAGATCAAGATCATTGCTCCT-3′

ACTIN: R 5′- TACTCCTGCTTGCTGATCCA-3′

KU80: F 5′- GCACTGACAATCCCCTTTCTG-3′

KU80: R 5′- TCAATGTCCTCCAGCAAATCAAA-3′

KU70: F 5′- TTGCTTCTGCCTAGCGATACC-3′

KU70: R 5′- AAACCTGGATCATCAAACCGTT-3′

RAD 51: F 5′- CGAGCGTTCAACACAGACCA-3′

RAD51: R 5′- GTGGCACTGTCTACAATAAGCA-3′

BRCA 1: F 5′-TTGTTACAAATCACCCCTCAAGG-3′

BRCA1: R 5′-CCCTGATACTTTTCTGGATGCC-3′

NDUFS 1: F 5′-TTAGCAAATCACCCATTGGACTG-3′

NDUFS1: R 5′-CCCCTCTAAAAATCGGCTCCTA-3′

### Nuclear and cytoplasmic protein extraction

The commercially available kit: Nuclear and Cytoplasmic Extraction Reagents Kit was purchased from Thermo Fisher Scientific (no.78833). Briefly, adherent cells were harvest with trypsin-EDTA and then centrifuged at 500 × *g* for 5 min, then washed with PBS and transfer 3 × 10^6^ cells to a 1.5 mL microcentrifuge tube. Add 200ul ice-cold CER I to the tube, and vortex the tube vigorously on the highest setting for 15 s and incubated the tube on ice for 10 min. Add 11ul ice-cold CER II to the tube, then centrifuged the tube for 5 min at maximum speed, the supernatant was cytoplasmic protein. Next, add 100ul ice-cold NER, and vortex for 15 s every 10 min, for a total of 40 min. Centrifuge the tube at maximum speed for 10 min, the supernatant was nuclear protein.

### Western blot analysis

Cell extracts and were prepared with ice-cold 1 × RIPA buffer (Promoterbio) supplemented with protease and phosphatase inhibitors (MCE HY-K0010). Tumor tissues were prepared with ice-cold 1 × RIPA buffer (1 mg/1 ml) supplemented with protease inhibitors, was homogenized, and centrifuged 10,000 × *g* for 15 min at 4 °C to get the supernatants. Equals amount of protein extracts were separated in SDS-PAGE 7.5–12% Bis-Tris protein gels (EpiZyme) and transferred to PDVF membranes (Millipore). Immunoreactive bands were developed with the Chemiluminescence ECL Detection System (Advasta K-12045-D50), and signals were detected using the LAS-4000 Mini Luminescent Image Analyzer (Thermo Fisher Scientific). Statistical analysis of all western blot data was listed in Supplementary Fig. 5.

### Clone formation

Cells were used in a colony formation assay to measure cell survival after IR treatment using standard protocols. Cells were plated, irradiated overnight at various doses, and allowed to grow for 7–14 d. Any colonies formed were fixed and stained with crystal violet (Biyotime C0121). The number of colonies (with >50 cells) per dish was counted, and the surviving fractions were calculated as the ratio of the plating efficiency of treated cells to untreated cells.

### Apoptosis assay

Cancer cells were seeded at 2 × 10^5^ cells/well in six-well plates overnight, next were subjected to irradiation with a range of doses (0, 4 Gy) for 48 h and then harvested by trypsinization. Quantification of apoptosis at the single-cell level was as described previously by a combination of flow cytometry using the YF®488-Annexin V and PI Apoptosis Kit (US EVERBRIGHT Y6002).

### Confocal microscopy

Cell lines grown on coverslips were fixed with 4% paraformaldehyde for 30 min at room temperature, permeabilized with 0.5% Triton X-100 for 5 min at 4 °C, and incubated with primary antibodies (γH2AX and PDH) overnight at 4 °C. The slides were then incubated with Alexa 488–conjugated (green; Invitrogen) or Alexa 594–conjugated (red; Invitrogen) secondary antibodies for 2 h at 4 °C. The coverslips were washed with PBST, incubated with DAPI for 15 min, and mounted in 60% glycerol. Cells were seeded onto confocal small dish, then were loaded with mitochondria-selective Mitotracker Green (Thermo Fisher Scientific, Cat#M7512) for 15 min or Rhod-2AM (Thermo Fisher Scientific, Cat.R1245MP) for 40 min under normal culture conditions. then wash with HBSS. Images were captured with a confocal laser microscope (Leica TCS SP5 II). At least 100 cells were analyzed for each group.

### Xenograft tumor

All animal work was performed in accordance with the guidelines of the Institutional Animal Care and Use Committee of Wuhan University under approved protocols. Six-week-old female BALB/c nude mice were purchased from Center for Disease Control and Prevention of Hubei Province (Wuhan, China) and housed in a specific pathogen-free, temperature and humidity-controlled environment with food and water in their cages. In total, 6 × 10^6^ cells were washed in PBS and injected subcutaneously into female BALB/c nude mice (*n* = 5) after group randomization to study the function of ndufs1. X-ray radiation therapy, 10 Gy/1 F was commenced (Supplemental Fig. 3c) when the tumor volume reached approximately 300 mm^3^. The mice were sacrificed when the following points were reached: (1) tumor size reached 1500 mm^3^; (2) the tumor surface appeared to have anabrosis. The tissues from the tumor-bearing mice were fixed in 4% PFA at 4 °C overnight and embedded into paraffin (Paraplast, Sigma-Aldrich) using tissue processor.

### IHC

Inclusion criteria: (1) Age is range 18–70 years old, gender is not limited; (2) The pathological diagnosis of CRC by biopsy in our hospital; (3) After diagnosis, neoadjuvant radiotherapy and chemotherapy will be performed first, followed by radical surgery; (4) The radiotherapy site is the primary tumor; (5) The chemotherapy regimen is mainly based on mFOLFOX6 and XELOX; (6) The pathology report contains TRG score.

Exclusion criteria: (1) Receive internal radiation therapy; (2) Received radiotherapy before and the radiation field may involve the colorectal site.

The criteria for TRG are: Grade 0 (No viable cancer cells, complete response); Grade 1 (Single cells or rare small groups of cancer cells, near-complete response); Grade 2 (Residual cancer with evident tumor regression, but more than single cells or rare small groups of cancer cells, partial response); Grade 3 (Extensive residual cancer with no evident tumor regression, poor or no response).

Paraffin sections were cut to 5-μm thickness, deparaffinized with xylene, and rehydrated with graded ethanol. NDUFS1, PDH, or HIF-1α staining was performed after antigen retrieval with mM EDTA (PH:8), 10 mM citrate buffer, or 1 mM EDTA plus 10 mM Tris-Cl (PH:8), respectively. Sections were washed three times in PBS, treated with 3% H_2_O_2_ in PBS for 15 min, blocked in 10% goat serum and 0.3% Triton X-100 in PBS for 1 h, and incubated with primary antibody against ndufs1, ndufa9, or pdha1 overnight, followed by incubation with secondary horseradish peroxidase, alkaline phosphatase, or biotin-conjugated Abs (Jackson Immuno Research Laboratories and Vector Laboratories) for 2 h. Samples were then incubated with an EnVision Dual Link (Dako) secondary antibody for 30 min. Samples were visualized with diaminobenzidine (Dako), counterstained with hematoxylin, dehydrated in ethanol, and cleared in xylene. Negative controls (substitution of the primary antibody with TBS) were run simultaneously.

### Digital image analysis

IHC tissue sections were converted into digital format at ×20 magnification with a Scanscope CS device (Aperio) and annotated with Digital Image Hub software (Slide Path). Annotated regions were subjected to image analysis with Tissue IA version 3.0 software (Slide Path). NDUFS1 (1:400 in CRC, 1:1000 in cervical cancer), NDUFA9 (1:300), and PDH (1:4000) expression on the basis of intensity and stained tissue area were quantified by pathologists, who were blinded to the group allocation. These parameters were combined to assign an IHC score of 0–15 to each sample, representing minimal to maximal levels of expression respectively.

### Statistics

All data are representative of at least three separate experiments. The results were analyzed using PRISM software (GraphPad Software Inc, USA). Variance was similar between the groups, and group differences were analyzed using a two-tailed Student’s *t* test, *P* < 0.05 was considered significant difference. Survival distributions were estimated using the Kaplan–Meier method with the log-rank test. All data were representative of multiple independent experiments.

## Supplementary information

supplemental Figure Legends

S1

S2

S3

S4

S5
