# Supplementary material for: Mitochondrial dysfunction induces radioresistance in colorectal cancer by activating [Ca2+]m-PDP1-PDH-histone acetylation retrograde signaling
Source: Cell Death Dis. 2021 Sep 6;12(9):837. doi: 10.1038/s41419-021-03984-2 (PMC8421510; doi:10.1038/s41419-021-03984-2)
Supplement: Supplementary file 1 — supplemental Figure Legends [file 41419_2021_3984_MOESM1_ESM.docx]

Supplemental Figure Legends

fig-1. Kaplan-Meier curves showing the overall survival in CRC cases with low or high mitochondrial complex I gene (NDUFA10, NDUFV2, NDUFS3, NDUFV3) expression from TCGA databases, and disease free survivals in CRC patients with low or high NDUFA10, NDUFV2 from TCGA databases.

fig-2. **a,** The proliferation of NC and ROT cells. (n = 4), ***P* < 0.01. **b,** Apoptosis in NC and ROT cells with or without 10μM oxaliplatin for 24h. (n = 3), ***P* < 0.01. **c,** the left is the ROS level in NC, ROT and ROT+NAC (5mM) groups, the right is the apoptosis in ROT and ROT+NAC (5mM) cells with or without 4Gy X-ray irradiation. (n = 3), ***P* < 0.01. Data are presented as the means ± s.e.m. **d,** The relationship between mitochondrial Ca^2+^ uptake pathway and ΔΨm.

fig-3. **a-b,** The protein and mRNA levels of NDUFS1 gene in ROT-blank、ROT-ndufs1/ctrl、ROT-ndufs1/oe cells. (n = 4), ***P* < 0.01. **c,** Simulation positioning before irradiation. **d,** The proliferation of ROT-ndufs1/ctrl and ROT-ndufs1/oe cells. Data are presented as the means ± s.e.m.

fig-4. **a,** Kaplan-Meier curves showing the disease free survivals in CRC cases with low or high PDK1 expression from TCGA databases. **b,** IHC staining of a tissue microarray of 84 Cervical cancer samples for NDUFA9. Representative images are shown of low and high staining. Kaplan-Meier survival analysis of NDUFA9 expression in 84 Cervical cancer patients divided into two group. Higher NDUFA9 expression is related to better radiotherapy efficacy.

fig-5. **a,** Relative protein levels of complex I genes in Fig2B were normalized to ACTIN (n = 3). **b,** Relative protein levels of DNA repair genes in Fig2G were normalized to ACTIN (n = 3). **c,** Relative protein levels of PDH in cytoplasm were normalized to ACTIN, in nucleus were normalized to H3 in Fig3F and Fig4I (n = 3). **d,** Relative protein levels of H3K56ac, H3K9ac were normalized to H3, others were normalized to ACTIN in Fig3I (n = 3). **e,** Relative protein levels of PDP1, p-PDH, PDH were normalized to ACTIN in Fig4D (n = 3). **f,** Relative protein levels of H3K56ac, H3K9ac were normalized to H3, others were normalized to ACTIN in Fig4J (n = 3). **g,** Relative protein levels of DNA repair genes in Fig5E were normalized to ACTIN (n = 3). **h**. Relative protein levels of HIF-1a, NDUFS1, PDH were normalized to ACTIN in Fig6H (n = 3). P value was calculated by one-way ANOVA, **P* < 0.05, ***P* < 0.01, ***P < 0.001, ****P < 0.0001
