# Supplementary figures and images for: Mitochondrial dysfunction induces radioresistance in colorectal cancer by activating [Ca2+]m-PDP1-PDH-histone acetylation retrograde signaling
Source: Cell Death Dis. 2021 Sep 6;12(9):837. doi: 10.1038/s41419-021-03984-2 (PMC8421510; doi:10.1038/s41419-021-03984-2)

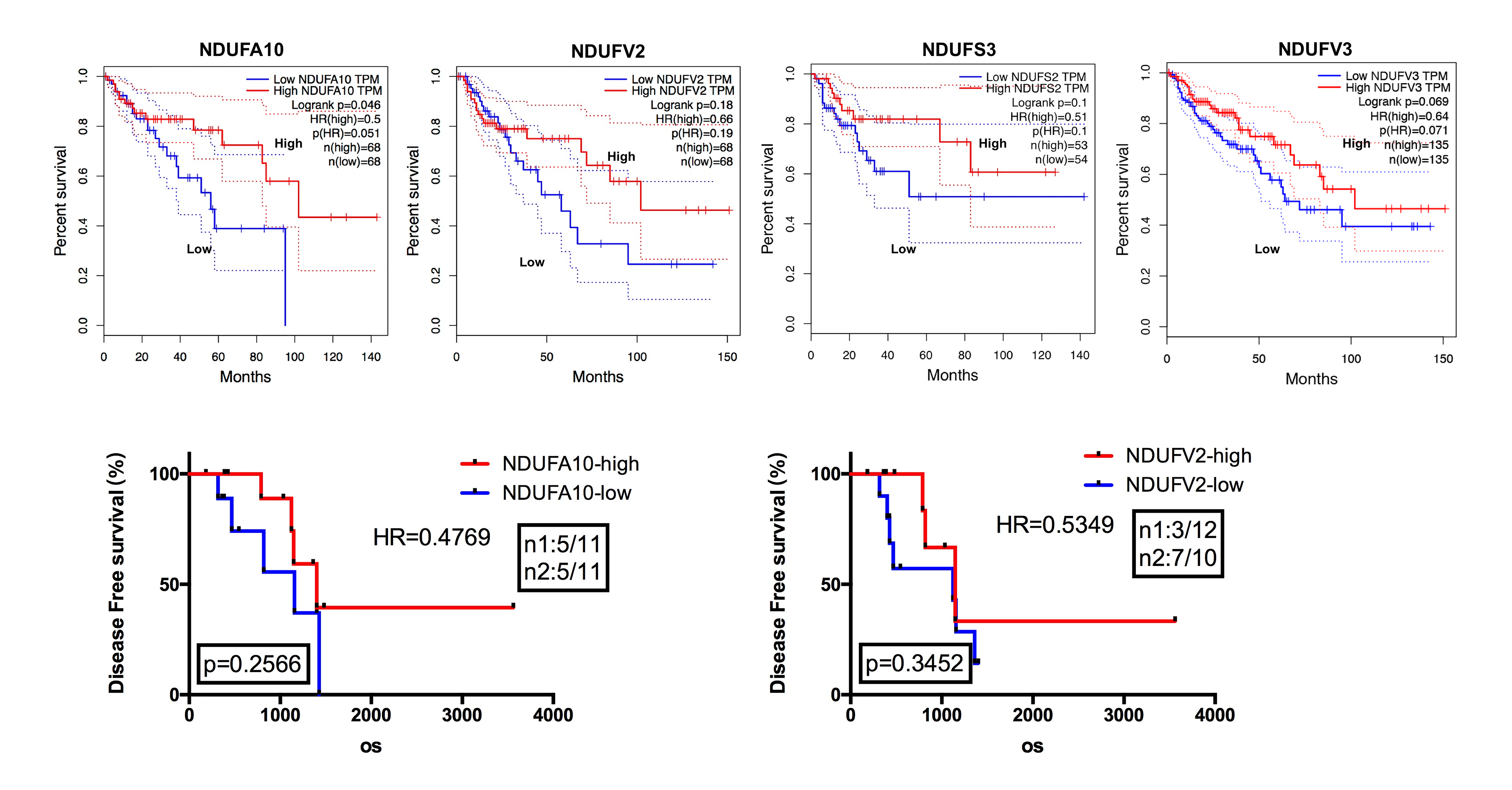

Supplement: Supplementary file 2 — S1 [file 41419_2021_3984_MOESM2_ESM.png]

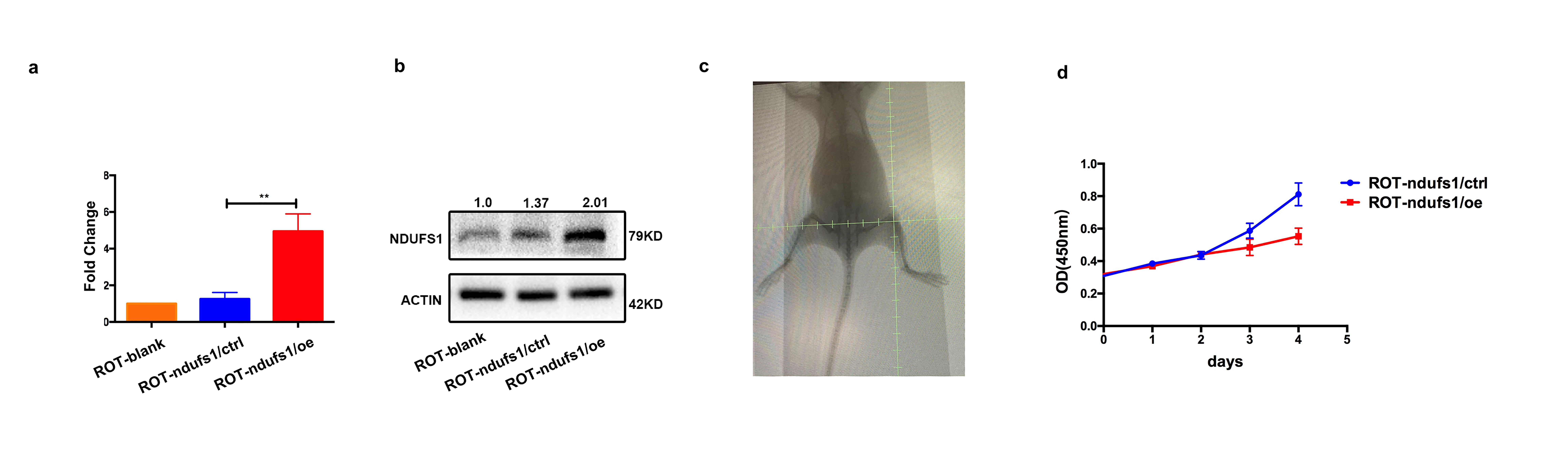

Supplement: Supplementary file 4 — S3 [file 41419_2021_3984_MOESM4_ESM.png]

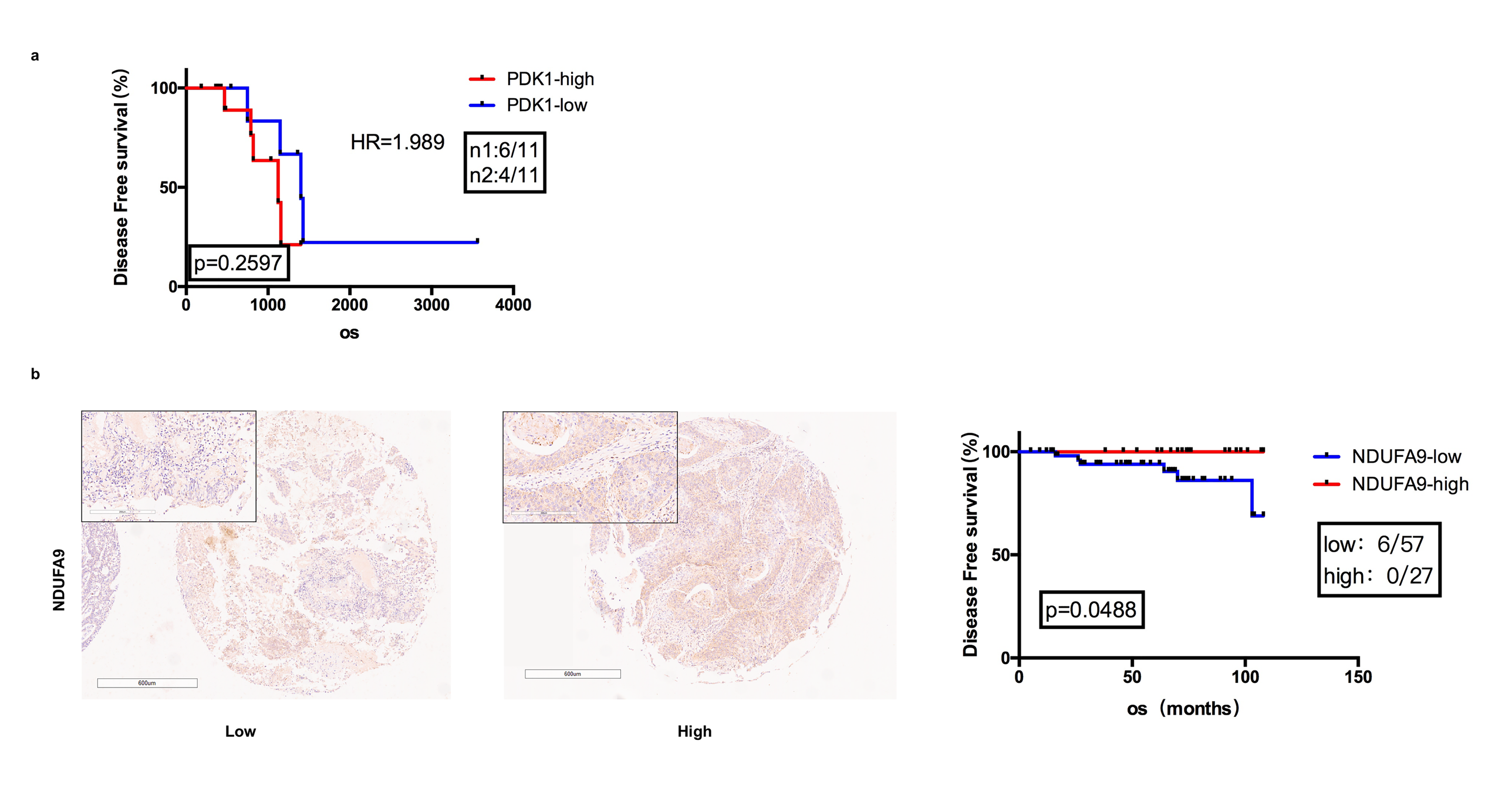

Supplement: Supplementary file 5 — S4 [file 41419_2021_3984_MOESM5_ESM.png]
